# Angle Closure Glaucoma in Retinitis Pigmentosa

**DOI:** 10.1155/2020/6023586

**Published:** 2020-05-29

**Authors:** Chandni Pradhan, Simanta Khadka, Purushottam Joshi

**Affiliations:** ^1^Mechi Eye Hospital, Birtamod-9, Jhapa, Nepal; ^2^Bharatpur Eye Hospital, Bharatpur-10, Chitwan, Nepal

## Abstract

**Background:**

Angle closure glaucoma (ACG) whether primary or secondary lens induced has rare occurrence in cases with retinitis pigmentosa (RP).

**Method:**

Five patients with history of diminished vision, ocular pain, and nyctalopia were clinically evaluated. Four patients had unilateral presentations of circumciliary congestion, corneal edema, and high intraocular pressure (IOP), while one had bilateral presentation, respectively. Anterior chambers were shallow; fundoscopy revealed the features of RP and gonioscopy affirmed closed angles in all the cases. The management strategies were individualized based on the specific ocular condition.

**Result:**

The raised IOP were not well controlled with conventional medical treatment. Neodymium yttrium aluminium garnet laser peripheral iridotomy (LPI) was performed in two patients and in the fellow eye in other two patients as a prophylactic measure. Phacoemulsification surgery with implantation of intraocular lens (IOL) was performed in three patients, whereas phacoemulsification only without IOL and trabeculectomy performed in one patient. Among them, two patients had subluxated lens, where one was managed with capsular tension ring and the other was left aphakic, respectively. However, the vision was not improved significantly in these patients.

**Conclusion:**

RP may be associated with ACG in rare instances. In these patients, angle closure-related high IOP can have a detrimental effect on the pre-existing visual impairment. However, this can be prevented by thorough clinical examination and timely intervention in those susceptible eyes.

## 1. Introduction

Retinitis pigmentosa (RP) is the term used for a diverse group of progressive hereditary disorders that primarily affect photoreceptors and retinal pigment epithelial (RPE) function. It predominantly affects the rods, followed by subsequent degeneration of cones [1]. The association between RP and glaucoma has been long sought. The first case of RP associated with glaucoma was described by Galezowski in 1862 [2]. Since then there have only been few reports of glaucoma associated with RP. The prevalence of primary open-angle glaucoma with RP ranges from 2 to 12% [3–5]. However, the association of RP and primary angle-closure glaucoma (PACG) has rarely been reported. Badeeb et al. reported the prevalence of 1.03% PACG in RP in patients over 40 years of age [3]. In view of this rarity, the association might be coincidental [4].

Intraocular pressure (IOP) elevation, due to acute angle closure may aggravate the visual impairment in RP patients with pre-existing optic nerve dysfunction [5–7]. Angle closure-related IOP elevation can be prevented by timely intervention in these susceptible eyes. Therefore, understanding the association of angle closure and RP may help preserve visual function in these patients.

Here, we have reported five unusual cases of angle-closure glaucoma (ACG) in RP along with their case-based management. Informed consents of the included patients were obtained, and all the work conducted were in accordance with the Declaration of Helsinki (1964).

## 2. Case Description

### 2.1. Case 1

**Figure 1:**
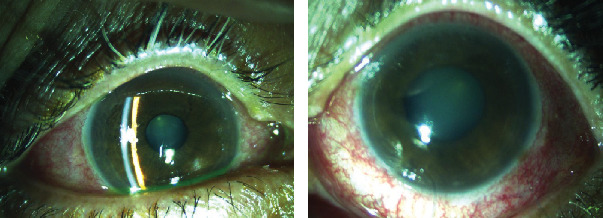
Clinical photograph of anterior segment OD and OS of case 1.

**Figure 2:**
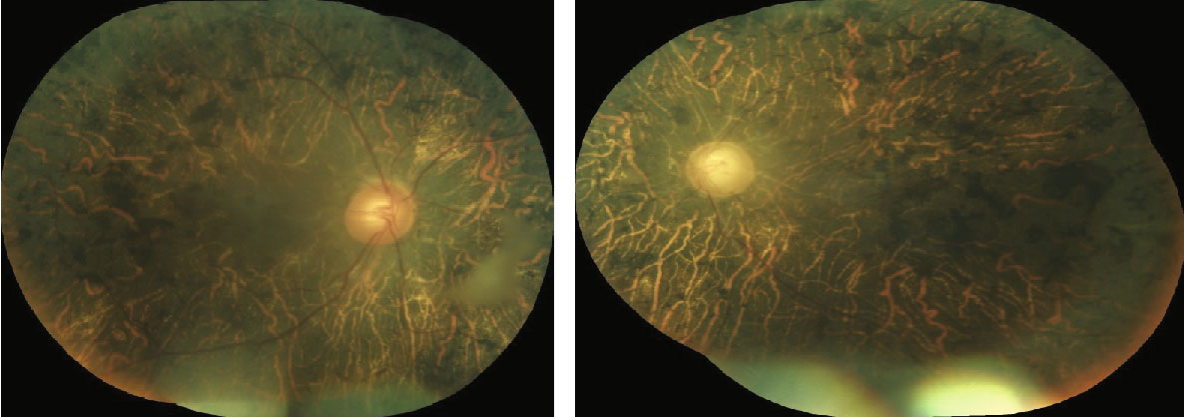
Fundus photo OD and OS of case 1.

A 65-year-old female, with a history of nyctalopia presented with a sudden, profound, and painful loss of vision OU of three days duration. She recounted a history of poor vision OU since 20 years. On examination, her visual acuity (VA) was hand movement (HM) OU. There was circumciliary congestion (CCC) with corneal edema (Figure 1). The anterior chambers were shallow with Van Herrick (VH) grade two. The pupil was middilated and sluggishly reacting to light OS and presence of posterior subcapsular cataract (PSCC) OU. Goldmann applanation tonometry (GAT) revealed an intraocular pressure (IOP) of 24 mmHg OD and 58 mmHg OS. She was managed with full tolerated antiglaucoma medications (Tab acetazolamide 250 mg, Gtt pilocarpine nitrate 2%, Gtt timolol maleate 0.5%+Gtt brimonidine tartrate 0.1%, and Gtt latanoprost 0.005%) and topical steroids (Gtt prednisolone acetate 1%). The following day, the corneal clarity was enhanced and her IOP was within a normal range of 11 mmHg OD and 12 mmHg OS, respectively. Gonioscopic examination disclosed closed angles in superior, temporal, and nasal quadrants OD, whereas, there was peripheral anterior synechiae (PAS) in all quadrant OS. Fundus examination revealed the cup disc ratio (CDR) 0.5 OD and 0.9 OS. The peripheral vessels were narrow and attenuated OU. There was diffuse RPE atrophy and bony spicules in the posterior pole and midperipheral retina (Figure 2). Neodymium yttrium aluminium garnett (Nd: YAG) laser peripheral iridotomy (LPI) was done OU followed by phacoemulsification with posterior chamber intraocular lens (PCIOL) OD. Her postoperative best corrected visual acuity (BCVA) was 4/60 OD, and IOP was regulated under control OU with topical medications and hence continued.

### 2.2. Case 2

**Figure 3:**
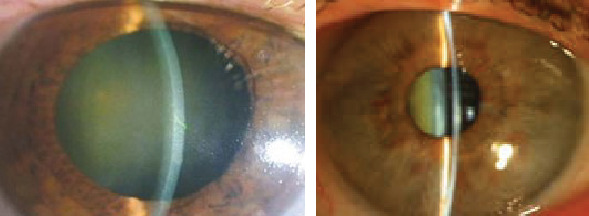
Clinical photograph of anterior segment OD and OS of case 2.

**Figure 4:**
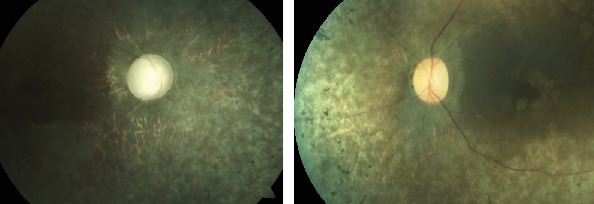
Fundus photograph OD and OS of case 2.

A 55-year-old female with a history of nyctalopia presented with painful loss of vision in RE since two weeks. She had no significant systemic illness or family history of ocular diseases. On examination, VA was HM OD and 6/18 OS. Congestion with corneal edema was evident with shallow AC (VH grade 1), middilated sluggishly reacting pupil, and presence of glaucomflecken with nuclear sclerosis (NS) grade 2 OD. Similarly, AC was also shallow (VH grade 2) and lens opacification of NS grade 2 OS (Figure 3). IOP with applanation was 46 mmHg OD and 17 mmHg OS. The patient was managed with full tolerated antiglaucoma medications. The following day, IOP was persistently high 38 mmHg OD despite medications, but the cornea was relatively clear allowing gonioscopy and fundus examination. On gonioscopy, there was presence of PAS in all quadrants OD. However, the angles were barely visible on indentation OS. On fundus examination, there was waxy pale discs OU, with near total cupping OD and CDR of 0.4 : 1 OS. The vessels were attenuated, and bony spicules diffusely distributed in peripheral retina OU (Figure 4). Prophylactic Nd:YAG LPI was done OS and lens extraction with PCIOL OD. The VA did not improve OD, but IOP declined to 22 mmHg one month past surgery. Antiglaucoma medications were continued OD.

### 2.3. Case 3

**Figure 5:**
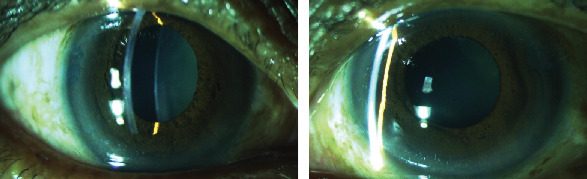
Clinical photograph of anterior segment OD and OS of case 3.

**Figure 6:**
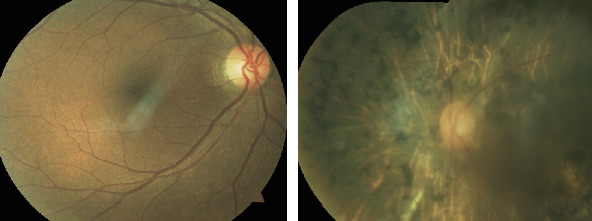
Fundus photograph of case 3 with normal looking posterior pole OD and presence of diffuse bony spicules OS.

A 62-year-old female presented with a history of painless loss of vision OS since 20 years. There was no significant family history. On examination, her BCVA was 6/36 OD and fingers counting close to face (FCCF) OS. Cornea was clear and AC shallow (VH grade 2) OU (Figure 5). Relative afferent pupillary defect (RAPD) was demonstrated during pupillary examination OS. There was nuclear sclerosis (NS) grade 2 OU. Gonioscopy divulged closed angles OD and presence of PAS in superior and temporal quadrants OS with IOP of 12 mmHg OD and 14 mmHg OS. On fundus examination, CDR was 0.3 : 1 OU. Peripheral retina was within normal limits OD, but there was presence of diffuse bony spicules OS (Figure 6). The patient mentioned inability to follow up as she was from a very distant area. LPI was done OU, and patient was planned for cataract surgery, but the patient declined.

### 2.4. Case 4

**Figure 7:**
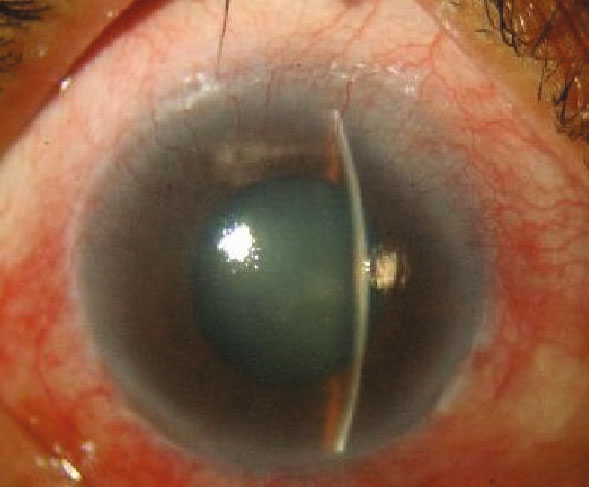
Anterior segment OS of case 4.

A 44-year-old male had a history of diminished vision OU (OS more marked than OD) since 14 years. He complained of pain and redness OS since one month. On examination, VA was 5/60 OD and HM OS. Anterior segment evaluation revealed shallow AC OD, while there was diffuse corneal edema with shallow AC, middilated pupils OS (Figure 7), and lenticular opacification of NS grade 2 OU. IOP was 16 mmHg OD and 65 mmHg OS, respectively. With commencement of maximum tolerated antiglaucoma medications, the cornea OS cleared allowing gonioscopy which revealed PAS in >270°. On fundus examination, discs were pale OU with CDR of 0.6 : 1 OD and 0.9 : 1 OS, respectively. Other than waxy pallor, the components of classic triad of RP were fulfilled with the presence of attenuated vessels and diffuse bony spicules OU.

Combined cataract surgery and trabeculectomy was planned OS. However, subluxated lens of three-clock hours from 6 to 9 o′clock were noted intraoperatively and implantation of capsular tension ring with PCIOL was possible in the bag at the conclusion of surgery. Prophylactic LPI was done in the fellow eye.

### 2.5. Case 5

**Figure 8:**
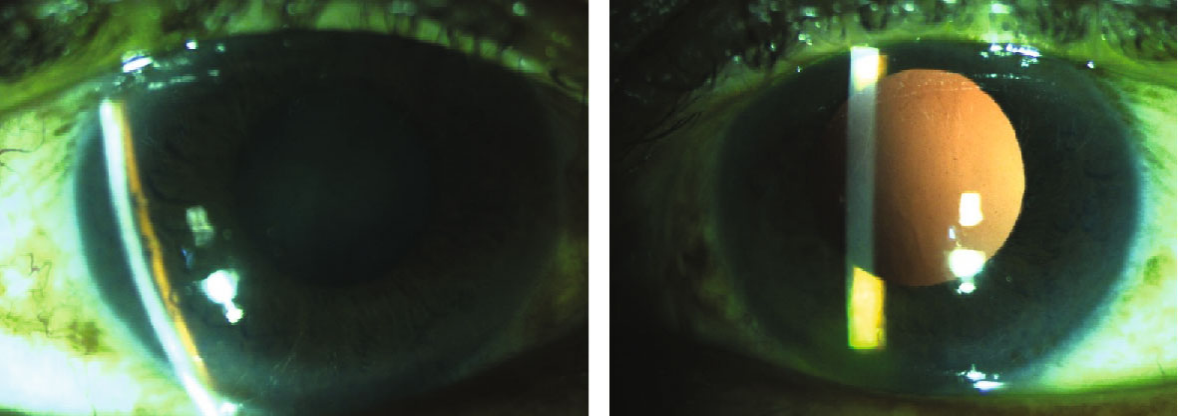
Clinical photograph of anterior segment OD and OS of case 5.

**Figure 9:**
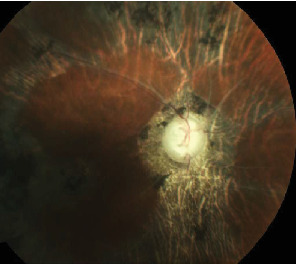
Fundus OD of case 5 examined after lens extraction surgery.

A 56-year-old male with complains of nyctalopia and diminished vision since three years. VA was perception of light with inaccurate projection of rays OD; however, there was no perception of light (NPL) OS. There was CCC, corneal edema OD with shallow AC OU. There was grade 3 NS OD, while the fellow eye was aphakic (Figure 8). The pupils were middilated and sluggishly reacting OU. The fundus visibility was very poor OD due to corneal edema and dense NS, and there was posteriorly dislocated lens in vitreous OS. Gonioscopy revealed PAS in three quadrants OS, but hazy media precluded angle evaluation OD. However, the patient denied history of trauma. The IOP was 60 mmHg OD and 35 mmHg OS. The IOP was controlled with maximum tolerated antiglaucoma medications. Combined cataract surgery and trabeculectomy with mitomycin C was performed OD. Intraoperative, subluxation of lens more than 180° from 3 to 10 clock hours was discovered and thorough anterior vitrectomy was performed and the patient was left aphakic. Superior flap with surgical iridectomy was created at superior 12 o′clock position. Postoperative ocular findings were uneventful and revealed no vitreous in AC, but there was no improvement in VA after surgery. The fundus examination post surgery affirmed pale disc with narrow attenuated peripheral vessels and diffuse RPE changes with bony spicules and attenuated arteriole. The presence of sclerosed venules implied probable overlapping sequelae of veno-occlusive disease (Figure 9).

Table 1 represents the ocular biometric parameters of all the subjects included in this series.  Table 2 represents summary of the cases with regard to vision, IOP, and management.

## 3. Discussion

We have reviewed five patients of ACG in RP who had visited our hospital at different times between July 2016 and June 2018. Though the literature indicating association between the two conditions are meagre, there are few reports of PACG with RP [3–5]. Among 234 diagnosed cases of RP in our hospital during this period, five cases presented as ACG accounting for prevalence of 2.13% in our series. A prevalence of 1.03% PACG in RP was reported from Canada [3]. Similarly, a five-year study from China showed 2.3% of RP associated with glaucoma, where angle closure was more frequent than open-angle glaucoma [8].

All of our patients were above 40 years, and three were females. It is an established fact that ACG occur more commonly in females. Similar female preponderance was reported to be 54.7% [5] and 56.52% [9] for RP, respectively.

A-scan ocular biometric readings were only included in this series as ultrasonic biomicroscopy (UBM) is not available in our setup. It is also suggested that the simultaneous occurrence of nanophthalmos, angle-closure glaucoma, and pigmentary retinal dystrophy could be a new syndrome [10, 11]. However, none of our patients were nanophthalmic, and the average AL was 21.87 mm OD and 21.38 mm OS, respectively. The role of UBM, where available, can provide reliable information and imaging evidence to evaluate the status of the lens position and determine subluxation for clinical use [12].

In this series, two patients had subluxated lens which were identified intraoperatively. There have been reports of lens subluxation in RP, causing anterior luxation of cataractous lens leading to angle closure. It is contemplated that the ultrastructure of the lens in RP is altered, causing lens fibre disorganisation which may contribute to the instability of the lens with anterior displacement and narrowing of angle [13]. Recently, zonular instability is speculated to be the cause behind angle closure in RP patient [14].

Previous theories regarding glaucoma in RP suggest that the migration of pigments is a characteristic feature of retinitis pigmentosa and these pigments in the angle of the anterior chamber has been stressed as a possible etiologic factor in glaucoma [15]. However, not all RP have grave prognosis. Sectoral RP, unilateral RP, and autosomal dominant inherited RPs have very slowly progressive disease course or could even be static [16]. In our series, we encountered only one patient with unilateral presentation.

In patients with RP, the high IOP due to angle closure can cause more visual impairment. Hence, a proper clinical work up, applanation tonometry, gonioscopy and timely intervention in these RP patients could decrease the risk of more damage by the comorbidity of ACG and preserve the ambulatory vision in these susceptible cases.

## Figures and Tables

**Table 1:** Ocular biometric parameters of the patients.

S. no.	Age (years)	Gender	Anterior chamber depth (mm)		Axial length (AL) (mm)		Lens thickness (mm)		Central corneal thickness (*μ*m)	
			OD	OS	OD	OS	OD	OS	OD	OS
1	65	F	2.39	1.98	22.52	22.56	4.61	4.89	555	536
2	55	F	2.34	2.12	22.9	21.86	4.98	4.82	494	496
3	63	F	2.49	2.49	20.81	20.39	4.81	5.22	535	555
4	44	M	2.13	1.88	22.05	21.56	4.22	4.89	560	590
5	56	M	2.15	2.8	21.08	20.54	4.83	—	552	538
Mean	56.6 ± 8.3		2.3	2.25	21.87	21.38	4.69	4.95	539.2	543

**Table 2:** Summary of vision, IOP, and management of the cases.

Case no.	Presenting VA		Presenting IOP (mm of Hg)		Management	Final VA		Final IOP (mm of Hg)	
	OD	OS	OD	OS		OD	OS	OD	OS
1	HM	HM	24	58	OU:LPI, OD:Phaco+PCIOL	4/60	HM	11	12
2	HM	6/18	46	17	OD:Phaco+PCIOL, OS:LPI	HM	6/18	22	17
3	6/36	FCCF	12	14	OU:LPI	6/36	FCCF	11	12
4	5/60	HM	16	65	OD:LPI, OS:Phaco+CTR+PCIOL	5/60	HM	16	14
5	PL	NPL	60	35	OD:Phaco+Trab+MMC	PL	NPL	20	18

LPI: Nd:YAG laser peripheral iridectomy; Phaco: phacoemulsification; PCIOL: posterior chamber intraocular lens; Trab: trabeculectomy; MMC: mitomycin C.

## Data Availability

Data is available in the text in the form of tables.
